# Evaluation of appropriate follow-up after curative surgery for patients with colorectal cancer using time to recurrence and survival after recurrence: a retrospective multicenter study

**DOI:** 10.18632/oncotarget.25312

**Published:** 2018-05-22

**Authors:** Tomoki Yamano, Shinichi Yamauchi, Kiyoshi Tsukamoto, Masafumi Noda, Masayoshi Kobayashi, Michiko Hamanaka, Akihito Babaya, Kei Kimura, Chihyon Son, Ayako Imada, Shino Tanaka, Masataka Ikeda, Naohiro Tomita, Kenichi Sugihara, Japanese Study Group for Postoperative Follow-up of Colorectal Cancer

**Affiliations:** ^1^ Division of Lower GI Surgery, Department of Surgery, Hyogo College of Medicine, Hyogo, Japan; ^2^ Division of Colorectal Surgery, Department of Surgery, Tokyo Medical and Dental University, Tokyo, Japan

**Keywords:** colorectal cancer, curative surgery, follow-up, recurrence, survival

## Abstract

**Introduction:**

The follow-up schedule for colorectal cancer patients after curative surgery is inconsistent among the guidelines. Evaluation of time to recurrence (TTR) and survival after recurrence (SAR) may provide evidence for appropriate follow-up.

**Methods:**

We assessed 3039 colon cancer (CC) and 1953 rectal cancer (RC) patients who underwent curative surgery between 2007 and 2008. We evaluated the pre- and post-recurrent clinicopathological factors associated with TTR and SAR in each stage of CC and RC.

**Results:**

The recurrence rates of stages I, II, and III were 1.2%, 13.1%, and 26.3%, respectively, for CC, and 8.4%, 20.0%, and 30.4%, respectively, for RC. In CC patients, high carcinoembryonic antigen (CEA) level and lymphovascular invasion were independent predictors of short TTR. In RC patients, metastatic factors (liver metastasis in stage III) and venous invasion (stage III) were independent predictors of short TTR. The prognostic factors of SAR were age (stage II CC and stage III RC), female gender (stage III RC), high CEA level (stage II RC), histological type (stage III CRC), nodal status (stage III CC), recurrence within 1 year (stage III RC), M1b recurrence (stage II CRC), local recurrence (stage II CC), and no surgical resection after recurrence (stage II and III CRC).

**Conclusions:**

The follow-up schedule for stage I should be different from that for the other stages. We recommend that intensive follow-up is appropriate in stage III CC patients with undifferentiated adenocarcinoma or N2 nodal status, stage II RC patients with high preoperative CEA level, and stage III RC patients.

## INTRODUCTION

Colorectal cancer (CRC) is one of the most common malignancies worldwide [1]. CRC has been increasing annually in Japan, and was estimated to be the most common malignancy in 2016 and the second most common cause of cancer-related deaths in 2014 [2].

Recurrence rates vary among patients who receive curative surgery, with recurrence reported in about 30% of stage III patients, 15% of stage II patients, and <5% of stage I patients in Japan [3]. Adequate surveillance is therefore required to both reduce costs and improve survival. However, surveillance is generally provided for patients without any recurrence.

The usefulness of intensive surveillance with routine carcinoembryonic antigen (CEA) monitoring and computed tomography (CT) scans remains controversial. Although early detection of recurrence has been shown to improve survival, early detection using intensive surveillance did not always result in improved overall survival [4–9]. The guidelines of the Japanese Society for Cancer of the Colon and Rectum propose intensive surveillance, including CEA checks every 3 months and CT every 6 months for 3 years, regardless of clinical stage and risk of recurrence [10]. This reflects the intensive surveillance schedules recommended by the European Society for Medical Oncology, American Society of Clinical Oncology, and National Comprehensive Cancer Network (NCCN) guidelines, which recommended CEA checks every 3–6 months and CT every 6–12 months for 2–3 years, depending on the risk of the patient [11–15].

However, these surveillances are not strictly categorized by risk factors other than clinical stage. Information on time to recurrence (TTR) and survival after recurrence (SAR) may help to determine the appropriate follow-up schedule, and has previously been used to evaluate the usefulness of intensive follow-up in clinical trials and meta-analysis [3, 5, 7, 8].

In this study, we evaluated the clinicopathological factors predicting TTR and SAR in patients with colon cancer (CC) or rectal cancer (RC) during intensive follow-up after curative resection, to determine the factors influencing the appropriate follow-up schedule.

## RESULTS

### Differences in clinicopathological factors between CC and RC

We compared the clinicopathological factors between CC and RC (Table 1). Age group, gender, preoperative CEA level, tumor depth, nodal status, venous invasion, application of adjuvant therapy, and clinical stage all differed significantly between the two locations.

**Table 1:** Clinicopathological factors of patients by tumor location

Location	CC	RC	*P* value	CC		RC		*P* value
Factors	(N=3039) Number (%)	(N=1953) Number (%)	CC vs RC	Recurrence rate	*P* value	Recurrence rate	*P* value	CC vs RC
Age								
≤64	1041 (34)	998 (51)	<0.0001	15.4%	NS	19.3%	NS	0.018
65–74	1090 (36)	629 (32)		13.1%		21.6%		<0.0001
75≤	908 (30)	326 (17)		13.4%		18.4%		0.03
Gender								
Male	1678 (55)	1192 (61)	<0.0001	14.8%	NS	21.4%	0.041	<0.0001
Female	1361 (45)	761 (39)		13.0%		17.6%		0.0044
Preoperative CEA								
High	831 (27)	561 (29)	0.043	23.9%	<0.0001	30.5%	<0.0001	0.0068
Normal	2115 (70)	1310 (67)		10.3%		15.3%		<0.0001
Unknown	93 ( 3)	82 (4)		9.7%		20.7%		0.04
Histological Type								
Well/Moderately	2867 (94)	1881 (96)	0.0069	13.6%	NS	19.2%	0.0002	<0.0001
Poorly	74 (2)	31 (2)		21.6%		41.9%	0.034	
Mucinous	98 (3)	41 (2)		18.4%		36.6%	0.021	
Tumor depth								
T1	592 (19)	355 (18)	<0.0001	1.0%	<0.0001	4.8%	<0.0001	0.0003
T2	447 (15)	438 (22)		3.6%		11.9%		<0.0001
T3	1411 (46)	971 (50)		14.0%		25.4%		<0.0001
T4	589 (19)	189 (10)		35.0%		38.6%		NS
Nodal status								
N0	2039 (67)	1256 (64)	<0.0001	7.9%	<0.0001	14.1%	<0.0001	<0.0001
N1	786 (26)	483 (25)		22.3%		26.3%		NS
N2	214 (7)	214 (119)		41.1%		39.7%		NS
Lymphatic invasion								
ly0	1323 (44)	793 (41)	NS	8.9%	<0.0001	13.1%	<0.0001	0.0023
ly1	1261 (41)	853 (44)		14.9%		21.3%		0.0001
ly2	378 (12)	262 (13)		23.5%		32.4%		0.013
ly3	58 (2)	37 (2)		48.3%		45.9%		NS
Unknown	19 (1)	8 (0)		10.5%		12.5%		NS
Venous invasion								
v0	1193 (39)	610 (31)	<0.0001	6.4%	<0.0001	10.5%	<0.0001	0.0025
v1	1188 (39)	765 (39)		15.3%		19.3%		0.021
v2	493 (16)	436 (22)		23.5%		30.0%		0.025
v3	140 (5)	125 (6)		32.1%		35.2%		NS
Unknown	25 (1)	17 (1)		20%		11.8%		NS
Stage								
I	890 (29)	641 (33)	<0.0001	1.2%	<0.0001	8.4%	<0.0001	<0.0001
II	1149 (38)	614 (31)		13.1%		20.0%		0.0002
III	1000 (33)	697 (36)		26.3%		30.4%		NS
Adjuvant therapy								
Stage I								
Yes	18 (2)	26 (4)	0.019	5.6%	NS	7.7%	NS	NS
No	872(98)	615 (96)		1.1%		8.5%		<0.0001
Stage II								
Yes	184 (16)	126 (20)	0.019	17.9%	0.036	25.4%	NS	NS
No	965 (84)	489 (80)		12.2%		18.6%		0.001
Stage III								
Yes	643 (64)	509 (73)	0.0002	25.8%	NS	31.0%	NS	NS
No	357 (36)	188 (27)		27.2%		28.7%		NS

CEA: Carcinoembryonic antigen, CC: colon cancer, NS: Not significant, Poorly: poorly differentiated adenocarcinoma, Mucinous: mucinous adenocarcinoma.

RC: rectal cancer, Well/Moderately: well differentiated/moderately differentiated adenocarcinoma.

### Factors associated with recurrence

The recurrence rates of stages I, II, and III CC were 1.2% (11/890), 13.1% (151/1149), and 26.3% (263/1000), and of RC were 8.4% (54/641), 20.0% (123/614), and 30.4% (212/697), respectively (Table 1). High preoperative CEA level, tumor depth, nodal status, lymphatic invasion, venous invasion, and clinical stage were significantly associated with recurrence, regardless of tumor location (Table 1). Gender and histological type were significantly associated with recurrence in RC, but not in CC.

We also assessed the influence of tumor location on recurrence in relation to each clinicopathological factor (Table 1). There were significant differences in recurrence rates between CC and RC for patients in each age group, with each CEA level, each histological type, and with tumor depth of T1-3, N0 nodal status, lymphatic invasion of ly0-2, venous invasion of v0-2, stage I-II and stage I-II without adjuvant therapy. However, there were no significant differences in recurrence rates by tumor location in relation to clinically-advanced factors including: depth of T4, N1 and N2 nodal status, lymphatic invasion of ly3, venous invasion of v3, and stage III.

### Factors associated with RFS and OS

**Figure 1:**
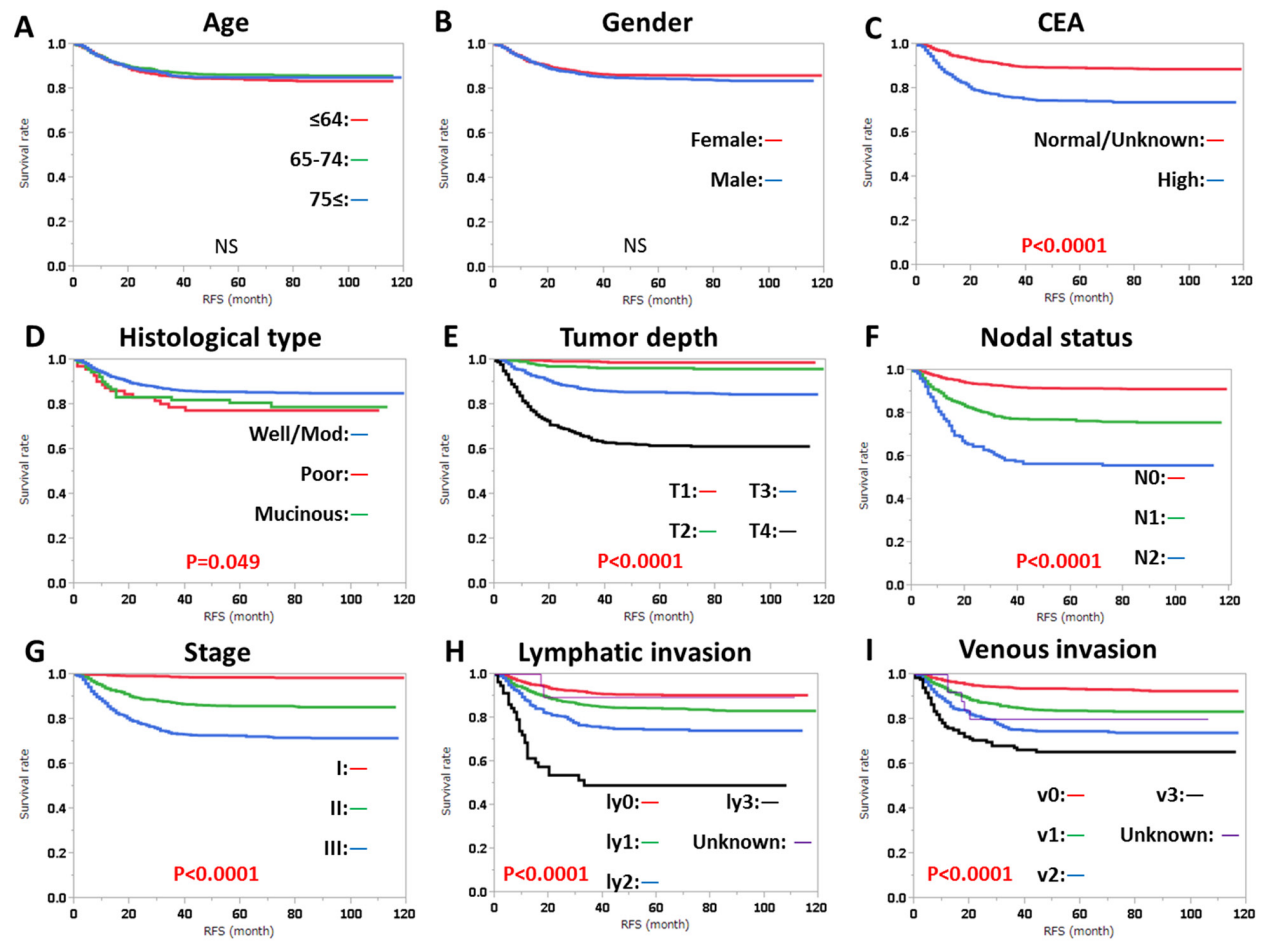
Relapse-free survival (RFS) in colon cancer according to **(A)** age, **(B)** gender, **(C)** CEA level, **(D)** histological type, **(E)** tumor depth, **(F)** nodal status, **(G)** clinical stage, **(H)** lymphatic invasion, and **(I)** venous invasion. Subgroups were compared with log-rank test. P values were provided when differences were significant (P<0.05) and as NS when differences were not significant.

**Figure 2:**
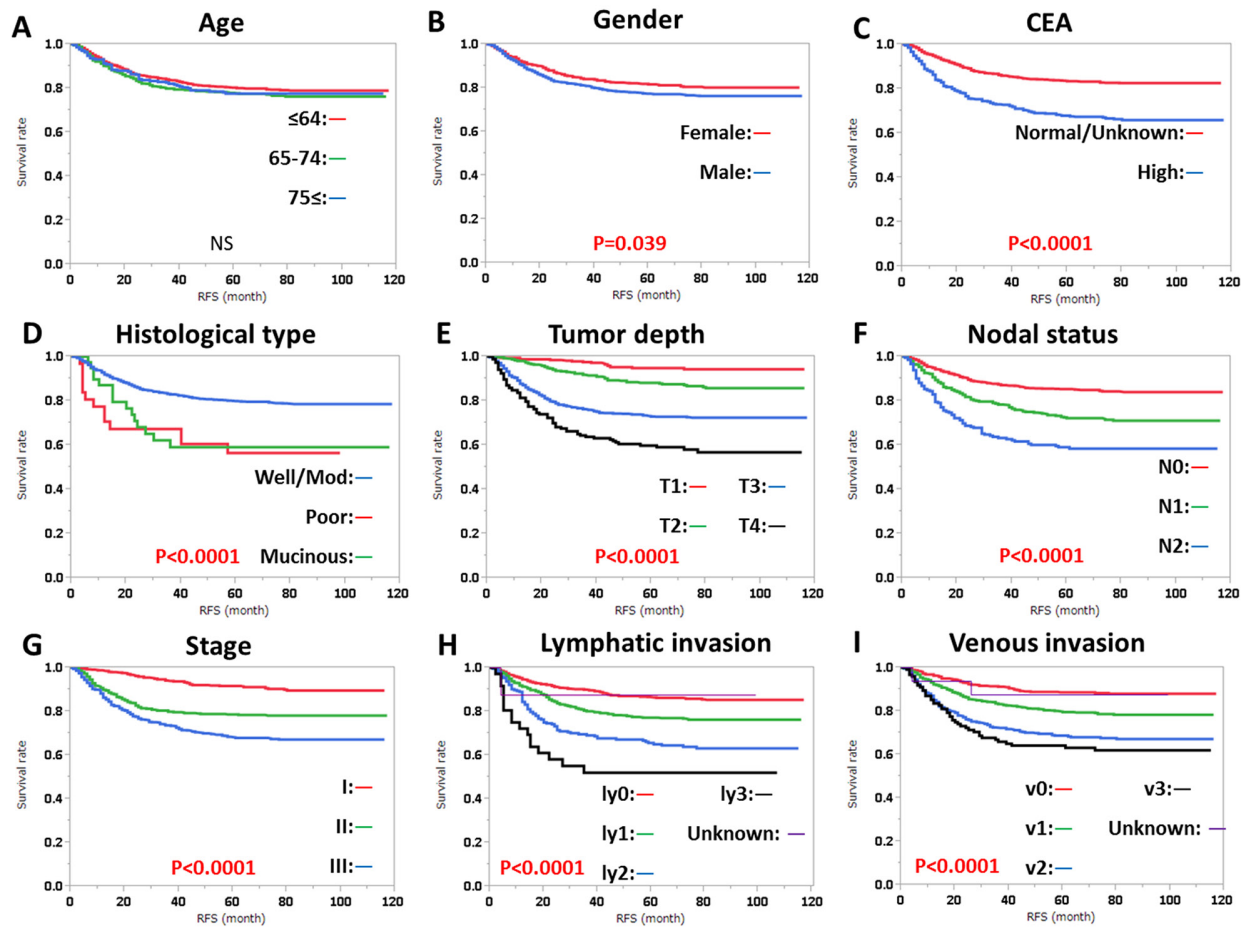
Relapse-free survival (RFS) in rectal cancer according to **(A)** age, **(B)** gender, **(C)** CEA level, **(D)** histological type, **(E)** tumor depth, **(F)** nodal status, **(G)** clinical stage, **(H)** lymphatic invasion, and **(I)** venous invasion. Subgroups were compared with log-rank test. P values were provided when differences were significant (P<0.05) and as NS when differences were not significant.

**Figure 3:**
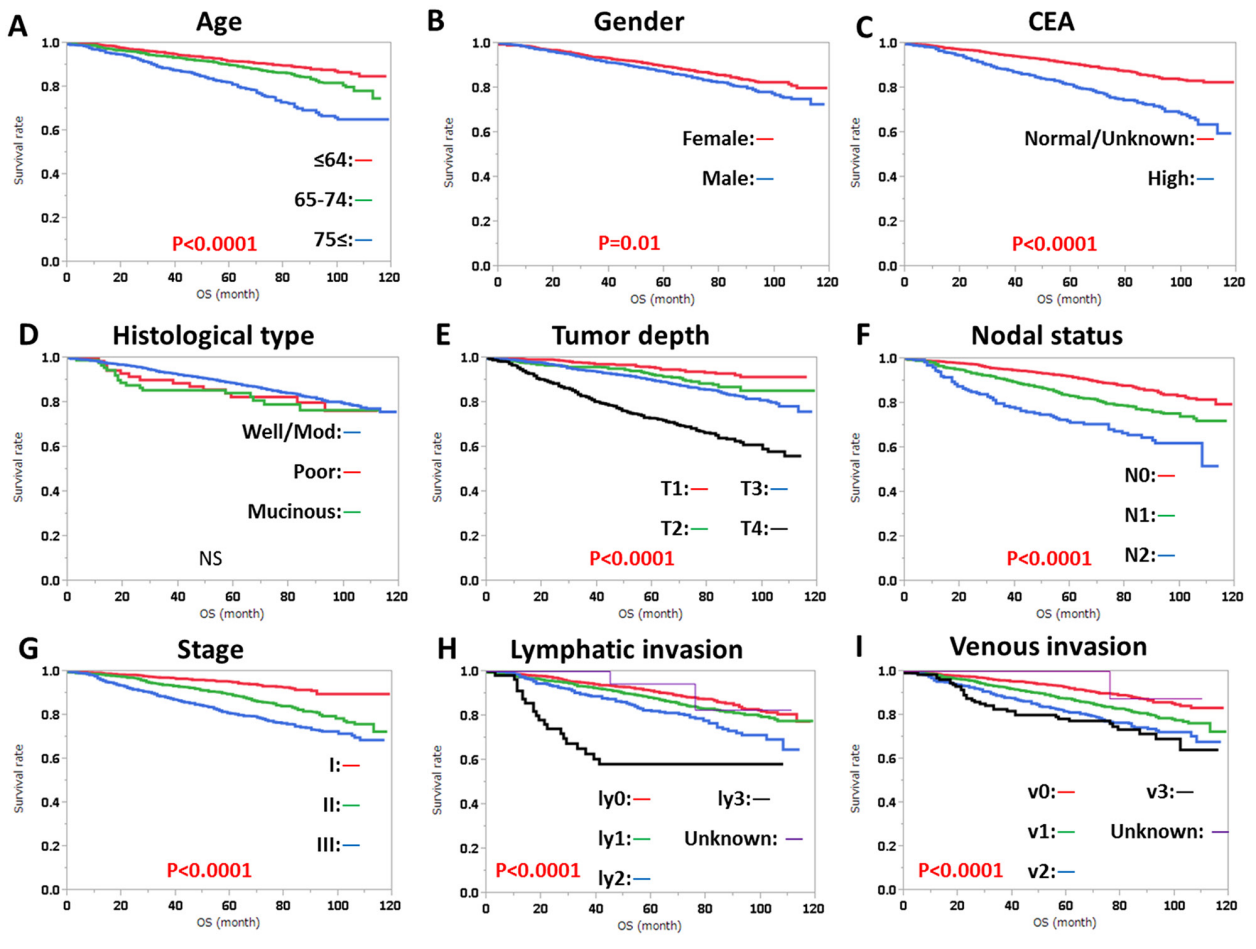
Overall survival (OS) in colon cancer according to **(A)** age, **(B)** gender, **(C)** CEA level, **(D)** histological type, **(E)** tumor depth, **(F)** nodal status, **(G)** clinical stage, **(H)** lymphatic invasion, and **(I)** venous invasion. Subgroups were compared with log-rank test. P values were provided when differences were significant (P<0.05) and as NS when differences were not significant.

**Figure 4:**
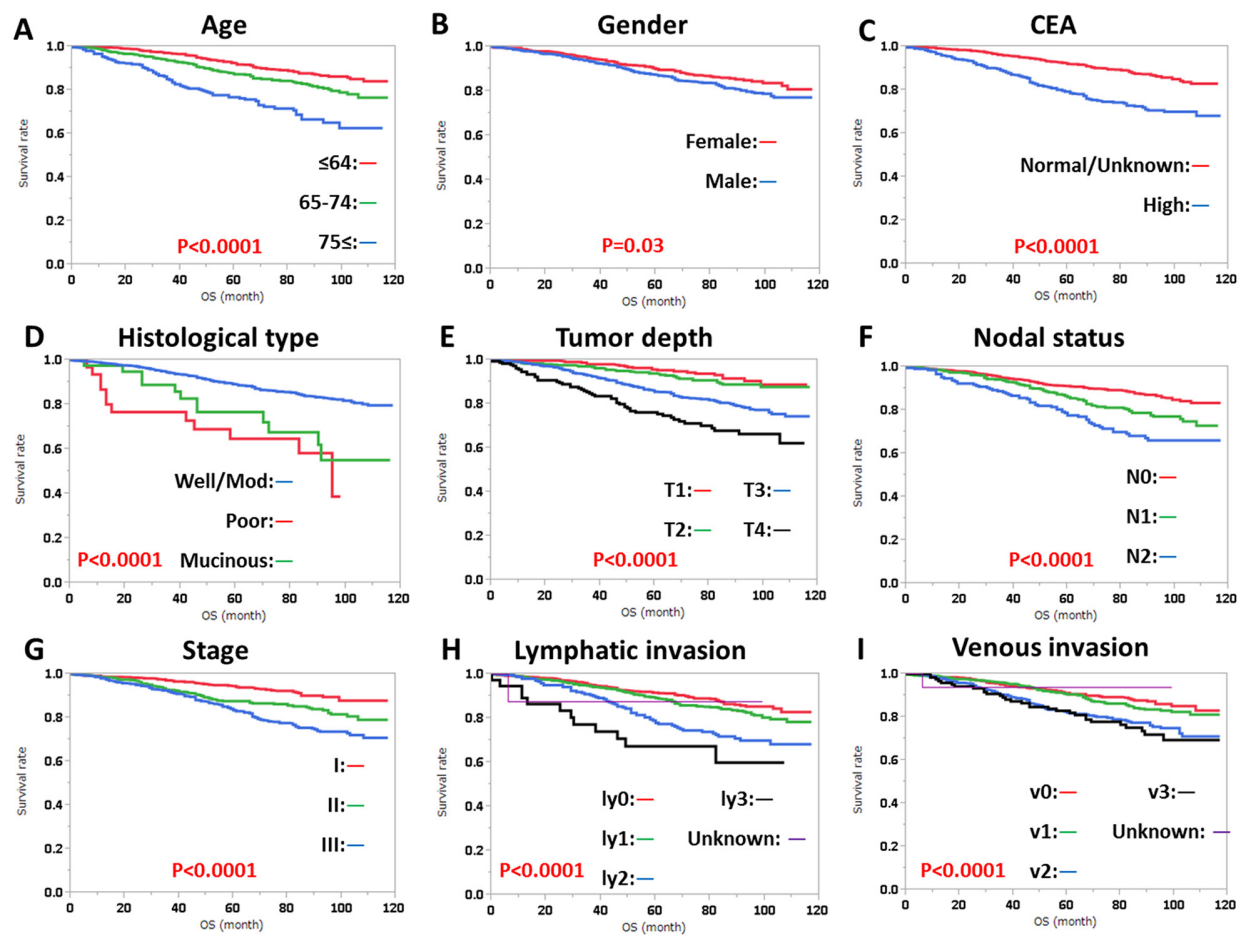
Overall survival (OS) in rectal cancer according to **(A)** age, **(B)** gender, **(C)** CEA level, **(D)** histological type, **(E)** tumor depth, **(F)** nodal status, **(G)** clinical stage, **(H)** lymphatic invasion, and **(I)** venous invasion. Subgroups were compared with log-rank test. P values were provided when differences were significant (P<0.05).

RFS was significantly influenced by preoperative CEA level, histological type, tumor depth, nodal status, stage, lymphatic invasion, and venous invasion, regardless of tumor location (Figures 1, 2). RFS was also significantly influenced by gender in patients with RC (Figure 2). OS was significantly influenced by age, gender, preoperative CEA level, tumor depth, nodal status, stage, lymphatic invasion, and venous invasion, regardless of tumor location (Figures 3, 4). OS was also significantly influenced by histological type in RC alone (Figure 4).

### Clinicopathological factors in patients with recurrence

Age group, gender, tumor depth, nodal status, stage, TTR, type of recurrence, and sites of recurrence differed significantly between CC and RC in patients with recurrence (Table 2). The liver was the most common metastatic site in CC patients, followed by the lungs, peritoneum, and distant lymph nodes. In contrast, the lungs were the most common metastatic site in RC patients, followed by the liver, local recurrence, and distant lymph nodes. There was no significant difference in preoperative CEA, histological type, or treatment after recurrence according to tumor location.

**Table 2:** Clinicopathological factors of patients with recurrence by tumor location

Factors / Location	CC (N=425) Number (%)	RC (N=389) Number (%)	*P* value CC vs RC
Age			
≤64 / 65–74/ 75≤	160/143/122 (38/33/29)	193/136/60 (50/35/15)	<0.0001
Gender			
Male / Female	248/177 (58/42)	255/134 (66/34)	0.035
Preoperative CEA			
High / Normal / Unknown	199/217/9 (47/51/2)	171/201/17 (44/52/4)	NS
Histological Type			
Well/Moderately / Poorly /Mucinous	391/16/18 (92/2/2)	361/13/15 (93/3/4)	NS
Tumor depth			
T1 / T2 / T3 / T4	6/16/197/206 (1/4/46/48)	17/52/247/73 (4/13/63/19)	<0.0001
Nodal status			
N0 / N1 / N2	162/175/88 (38/41/21)	177/127/85 (46/33/22)	0.034
Lymphatic invasion			
ly0 / ly1 / ly2 / ly3/Unknown	118/188/89/28/2 (28/44/21/7/1)	104/182/85/17/1 (27/47/22/4/0)	NS
Venous invasion			
v0 / v1 / v2 / v3 / Unknown	77/182/116/45/5 (18/43/27/11/1)	64/148/131/44/2 (16/38/34/11/1)	NS
Stage			
I / II / III	11/151/263 (3/36/62)	54/123/212 (14/32/54)	<0.0001
Time to recurrence (year)			
< 1 / 1–2/ 2–3 / 3≤	190/136/64/35 (45/32/15/8)	142/122/57/68 (37/31/15/17)	0.0007
Type of recurrence			
Local alone /M1a / M1b	29/251/145 (7/59/34)	77/243/69 (20/62/18)	<0.0001
Liver metastasis			
(+) / (-)	203/222 (48/52)	128/261 (33/67)	<0.0001
Lung metastasis			
(+) / (-)	130/295 (31/69)	148/241 (38/62)	0.025
Peritoneal metastasis			
(+) / (-)	83/342 (20/80)	28/361 (7/93)	<0.0001
Local metastasis			
(+) / (-)	41/384 (10/90)	112/277 (29/71)	<0.0001
Distant lymph node metastasis			
(+) / (-)	63/362 (15/85)	45/344 (12/88)	NS
Adjuvant therapy			
(+) / (-)	200/225 (47/53)	192/197 (49/51)	NS
Treatment after recurrence			
Best supportive care / Surgery (-) / Surgery (+)	38/188/199 (9/44/47)	30/167/192 (8/43/49)	NS

CEA: Carcinoembryonic antigen, CC: colon cancer, Mucinous: mucinous adenocarcinoma, NS: Not significant, Poorly: poorly differentiated adenocarcinoma, RC: rectal cancer, Well/Moderately: well differentiated/moderately differentiated adenocarcinoma

### Time to recurrence (TTR)

**Figure 5:**
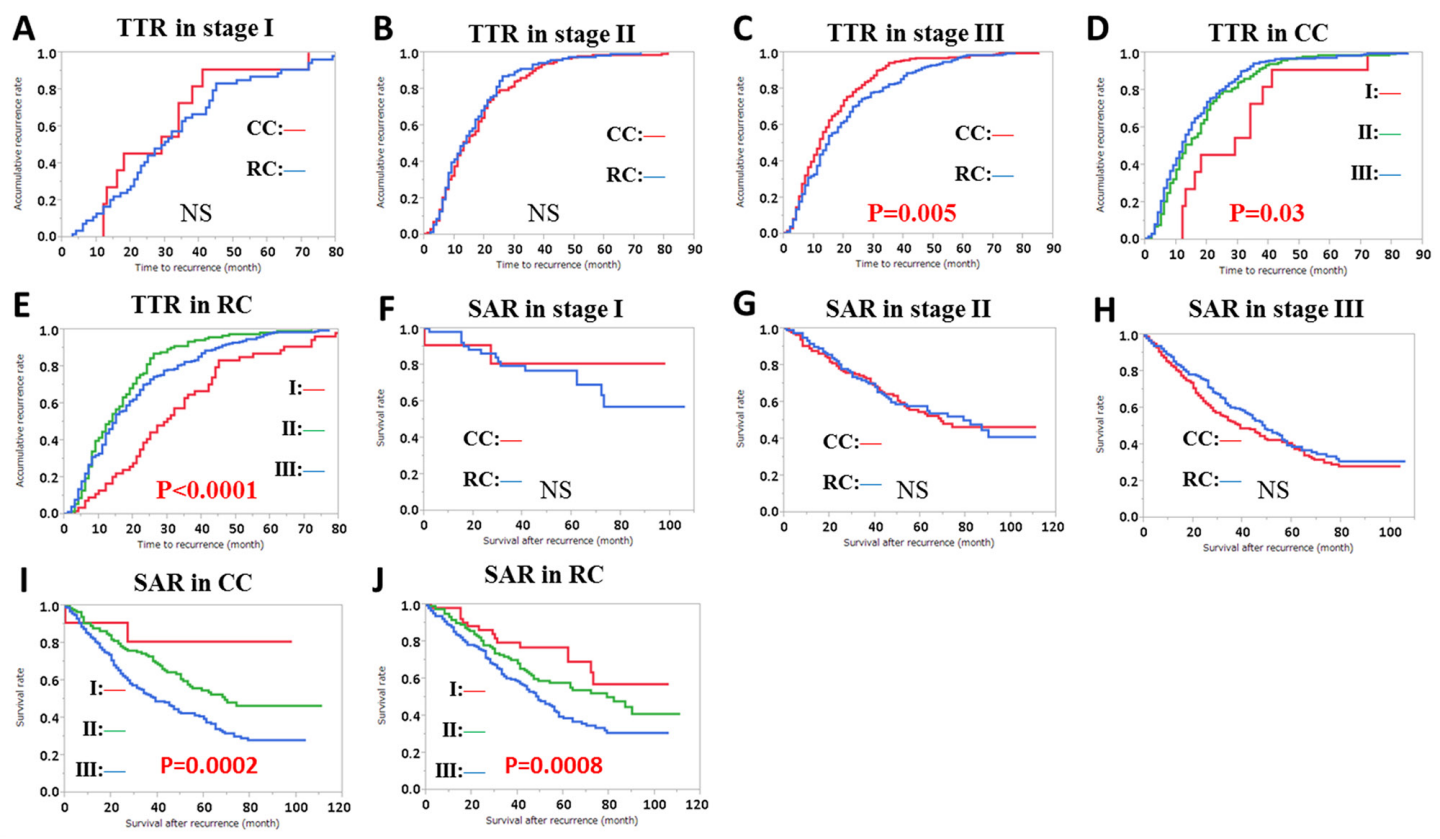
Time to recurrence (TTR) and accumulative recurrence rate **(A**-**E)**, and survival after recurrence (SAR) and survival rate **(F-J)**, by clinicopathological factors. TTR and accumulative recurrence rate in: (A) stage I patients by colon cancer (CC) and rectal cancer (RC), (B) stage II patients by CC and RC, (C) stage III patients by CC and RC, (D) CC by stage, (E) RC by stage. SAR and survival rate in: (F) stage I patients by CC and RC, (G) stage II patients by CC and RC, (H) stage III patients by CC and RC, (I) CC by stage, (J) RC by stage. Subgroups were compared with log-rank test. P values were provided when differences were significant (P<0.05) and NS was used when differences were not significant.

TTR was significantly longer in stage III patients with RC compared with CC patients, but not in stage I and II patients (Figure 5A-5C). The 1-, 2-, 3-, and 5-year accumulative recurrence rates in CC patients were 18%, 46%, 73%, and 91% for stage I; 46%, 78%, 90% and 99% for stage II; 53%, 80%, 94% and 97% for stage III, respectively (Figure 5D). The equivalent recurrence rates in RC patients were 17%, 41%, 65%, and 87% for stage I; 49%, 81%, 94%, and 98% for stage II; 44%, 71%, 83%, and 98% for stage III respectively (Figure 5E).

We further assessed TTR for CC and RC patients depending on stage, except for stage I, because there were fewer patients and longer TTR than for the other stages.

TTR in stage II CC was significantly associated with gender, preoperative CEA level, lymphatic invasion, and distant lymph node metastasis (Table 3). TTR in stage III CC was significantly associated with preoperative CEA level, lymphatic invasion, and venous invasion (Table 3). Multivariate analysis showed that high preoperative CEA level and lymphatic invasion were independent predictors for short TTR in both stage II and stage III CC (Table 4).

**Table 3:** Summary of associations between clinicopathological factors and TTR

Location	Colon cancer				Rectal cancer			
Stage	Stage II (N=151)		Stage III (N=263)		Stage II (N=123)		Stage III (N=212)	
Factors	Mean (months)	*P*	Mean (months)	*P*	Mean (months)	*P*	Mean (months)	*P*
Age								
≤74	17.5	NS	16.6	NS	16.2	NS	20.0	NS
75≤	18.0		13.9		17.4		19	
Gender								
Male	19.6	0.04	16.5	NS	16.9	NS	20.5	NS
Female	15.3		14.8		15.3		18.7	
Preoperative CEA								
High	14.7	0.02	14.1	0.04	16	NS	18.8	NS
Normal/Unknown	20.3		17.5		16.7		20.9	
Histological Type								
Well/Moderately	17.5	NS	16.0	NS	16.3	NS	20.2	NS
Poorly/Mucinous	20.5		14.1		18		16.7	
Tumor depth								
T1	-	NS	15.3	NS		NS	28.5	NS
T2	-		14.9				30.7	
T3	19.3		13.9		15.7		19.2	
T4	15.6		15.8		19.2		18.7	
Nodal status								
N0	17.7	-		NS	16.4	-		0.03
N1			16.3				21.8	
N2			14.9				16.9	
Lymphatic invasion								
ly0/1	18.5	0.008	16.7	NS	15.8	NS	16.7	NS
ly2/3	11.4		15.1		20.4		22.0	
ly0-2	17.8	NS	16.4	0.008	16.4	-	20.6	0.009
ly3	13		10.3		-		11.9	
Venous invasion								
v0/1	18.7	NS	17.2	NS	15.3	NS	22.4	0.02
v2/3	15.7		14.0		17.5		17.1	
v0-2	17.9	NS	16.6	0.001	16.3	NS	20.0	NS
v3	15.2		9.7		16.9		18.9	
Adjuvant therapy								
Yes	16.3	NS	16.9	NS	13.9	NS	19.7	NS
No	18.1		14.0		17.3		20.3	
Type of recurrence								
M1a	18.8	NS	15.5	NS	17.4	NS	21.0	0.02
M1b	15.0		15.2		13		14.8	
Local alone	20.5		23.4		15.7		22.9	
Liver metastasis								
(-)	18.6	NS	16.9	NS	18.6	0.01	23.0	<0.0001
(+)	16.8		14.5		13		12.8	
Lung metastasis								
(-)	17.2	NS	15.6	NS	15.8	NS	19.4	NS
(+)	18.5		16.2		17.6		20.4	
Peritoneal metastasis								
(-)	17.8	NS	16.2	NS	16.4	NS	20.2	NS
(+)	16.9		14.3		16		17.3	
Local recurrence								
(-)	17.6	NS	15.5	NS	16.8	NS	19.4	NS
(+)	18.1		18.9		15.4		21.1	
Distant LN metastasis								
(-)	18.3	0.04	15.2	NS	27.7	0.02	19.8	NS
(+)	11.9		18.4		15.5		20.4	

CEA: Carcinoembryonic antigen, LN: lymph node, Mucinous: mucinous adenocarcinoma, NS: Not significant, Poorly: poorly differentiated adenocarcinoma, TTR: time to recurrence, Well/Moderately: well differentiated/moderately differentiated adenocarcinoma.

**Table 4:** Multivariate analysis of predictors for short TTR

Location	Colon cancer				Rectal cancer			
Stage	Stage II		Stage III		Stage II		Stage III	
Factors	HR CI	*P*	HR CI	*P*	HR CI	*P*	HR CI	*P*
Gender: Male	0.73 0.53-1.02	NS						
CEA: High	1.47 1.06-2.03	0.02	1.34 1.04-1.73	0.02				
Nodal status: N2							1.23 0.92-1.64	NS
Lymphatic invasion								
ly2/3	1.83 1.03-3.05	0.04						
ly3			1.73 1.09-2.64	0.02			1.5 0.86-2.45	NS
Venous invasion								
v2/3							1.42 1.07-1.88	0.01
v3			1.63 1.09-2.36	0.02				
Type of recurrence:								
M1b							1.25 0.88-1.74	NS
Recurrence sites								
Liver (+)					1.44 0.99-2.09	NS	1.92 1.41-2.61	<0.0001
Distant LN: (+)	1.53 0.84-2.61	NS			0.52 0.23-1.03	NS		

CEA: Carcinoembryonic antigen, CI: confidence interval, HR: hazard ratio, LN: lymph node, NS: Not significant, TTR: time to recurrence.

TTR in stage II RC was significantly associated with metastatic factors (liver metastasis and distant lymph node metastasis), but not pathological factors. TTR in stage III RC was significantly associated with both pathological factors (lymphatic invasion and vascular invasion) and metastatic factors (M1b recurrence and liver metastasis) (Table 3). Multivariate analysis showed that venous invasion and liver metastasis were independent predictors for short TTR in stage III RC (Table 4).

### Survival after recurrence (SAR)

The 5-year survival rates after recurrence for CC and RC were 81% and 77% in stage I; 55% and 58% in stage II; 40% and 39% in stage III, respectively (Figure 5F–5H). There were significant differences in the 5-year SAR rates among stages, but not between CC and RC (Figure 5F–5J).

SAR was significantly associated with age (all groups), gender (stage III RC), CEA level (stage I/II RC), histological type (stage III CRC), tumor depth (CC), nodal status (stage III CC), lymphatic invasion (stage III CRC), adjuvant therapy (stage III RC), recurrence within 1 year (stage III RC), M1b recurrence (stage II/III CRC), liver metastasis (stage II RC), peritoneal metastasis (CC and stage III RC), local recurrence (stage II CC), and treatment after recurrence (all groups) (Table 5).

**Table 5:** Summary of associations between clinicopathological factors and SAR

Location	Colon cancer				Rectal cancer			
Stage	Stage II (N=151)		Stage III (N=263)		Stage II (N=123)		Stage III (N=212)	
Factors	Mean (months)	*P*	Mean (months)	*P*	Mean (months)	*P*	Mean (months)	*P*
Age								
≤74	59.1	0.0001	46.9	0.005	64.2	0.01	51.1	<0.0001
75≤	33.3		34.1		35.0		24.2	
Gender								
Male	51.9	NS	44.9	NS	59.5	NS	51.6	0.004
Female	51.9		41.5		64.7		39.6	
Preoperative CEA								
High	48.8	NS	44.9	NS	46.8	0.049	45.6	NS
Normal/Unknown	55.4		41.6		67.9		50.0	
Histological Type								
Well/Moderately	52.6	NS	45.8	0.003	61.8	NS	50.3	<0.0001
Poorly/Mucinous	44.5		18.4		17.4		23.8	
Tumor depth								
T1		0.009	16	0.002		NS	55	NS
T2			34.7				42.8	
T3	49.3		53.4		62.8		49.7	
T4	48.4		35.8		55.9		48.0	
Nodal status								
N1			47.1	0.02			50.3	NS
N2			36.4				41.5	
Lymphatic invasion								
ly0/1	51.4	NS	46.5	NS	63.2	NS	50.7	NS
ly2/3	49.6		38.7		37.2		40.7	
ly0-2	53.4	NS	45.5	0.004	61.6	-	49.2	0.005
ly3	14.7		16.3		-		29.6	
Venous invasion								
v0/1	51.5	NS	44.0	NS	65.8	NS	48.9	NS
v2/3	51.3		43.3		54.3		45.6	
v0-2	50.6	NS	44.3	NS	60.7	NS	48.3	NS
v3	58.1		38.8		53.7		44.2	
Adjuvant therapy								
Yes	57.0	NS	45.0	NS	65.5	NS	50.9	0.02
No	50.9		41.1		58.8		37.7	
Time to recurrence								
less than 1 year	48.9	NS	37.8	NS	58.1	NS	41.2	0.004
more than 1 year	53.0		48.1		62.9		50.4	
Type of recurrence								
M1a	59.0	0.0002	49.8	0.0001	61.2	0.001	53.6	0.0002
M1b	43.8		33.6		35.2		29.6	
Local alone	42.7		46.8		64.9		43.2	
Liver metastasis								
(-)	46.7	0.01	41.3	NS	60.4	NS	47.4	NS
(+)	57.8		46.3		48.9		48.4	
Lung metastasis								
(-)	56.0	NS	42.8	NS	65.9	0.02	46.0	NS
(+)	42.7		45.0		46.3		49.0	
Peritoneal metastasis								
(-)	54.2	0.03	47.8	<0.0001	62.5	NS	50.0	0.001
(+)	45.2		29.3		15.4		21.8	
Local recurrence								
(-)	54.1	0.04	44.1	NS	59.6	NS	48.3	NS
(+)	42.6		41.2		57.0		46.0	
Distant LN metastasis								
(-)	53.7	NS	44.5	NS	61.2	NS	49.4	NS
(+)	43.1		37.4		66.8		28.9	
Therapy after recurrence								
Surgical resection (-)	34.2	<0.0001	32.9	<0.0001	44.0	<0.0001	35.1	<0.0001
Surgical resection (+)	65.8		57.5		74.4		62.2	

CEA: Carcinoembryonic antigen, LN: lymph node, NS: Not significant, Mucinous: mucinous adenocarcinoma, Poorly: poorly differentiated adenocarcinoma, SAR: survival after recurrence, Well/Moderately: well differentiated/moderately differentiated adenocarcinoma.

Multivariate analysis identified the following independent prognostic factors: age (stage II CC and stage III RC), female gender (stage III RC), high CEA level (stage II RC), histological type (stage III CRC), nodal status (stage III CC), recurrence within 1 year (stage III RC), M1b recurrence (stage II CRC), local recurrence (stage II CC), and no surgical resection after recurrence (stage II and III CRC) (Table 6).

**Table 6:** Multivariate analysis of prognostic factors for SAR

Location	Colon cancer				Rectal cancer			
Stage	Stage II		Stage III		Stage II		Stage III	
Factors	HR CI	*P*	HR CI	*P*	HR CI	*P*	HR CI	*P*
Age: 75≤	2.71 1.56-4.67	0.0005	1.33 0.93-1.9	NS	1.77 0.81-3.56	NS	1.78 1.03-2.96	0.04
Gender: Female							1.69 1.14-2.47	0.009
CEA: High					2.33 1.34-4.11	0.003		
Histological type: Poorly/Mucinous			2.04 1.15-3.41	0.02			2.54 1.34-4.54	0.005
Tumor depth: T4	1.42 0.84-2.43	NS	1.32 0.93-1.87	NS				
Nodal status: N2			1.5 1.05-2.12	0.03				
Lymphatic invasion: ly3			1.23 0.66-2.15	NS				
Recurrence: within 1 year							1.64 1.12-2.4	0.01
Type of recurrence: M1b	2.50 1.25-4.83	0.01	1.39 0.84-2.18	NS	2.69 1.31-5.25	0.008	1.68 0.97-2.78	NS
Recurrence sites								
Liver (+)	1.04 0.58-1.84	NS						
Lung: (+)					1.25 0.68-2.24	NS		
Peritoneal: (+)	0.74 0.35-1.61	NS	1.37 0.82-2.36	NS			1.11 0.53-2.27	NS
Local: (+)	3.54 1.5-7.69	0.005						
No adjuvant therapy							1.00 0.64-1.53	NS
No surgical resection after recurrence	5.0 2.73-9.45	<0.0001	2.36 1.64-3.43	<0.0001	3.41 1.93-6.18	<0.0001	4.03 2.65-6.25	<0.0001

CEA: Carcinoembryonic antigen, CI: confidence interval, HR: hazard ratio, Mucinous: mucinous adenocarcinoma, NS: Not significant, Poorly: poorly differentiated adenocarcinoma, SAR: survival after recurrence.

## DISCUSSION

We evaluated the clinicopathological factors associated with TTR and SAR in patients with CC and RC, as critical factors for guiding appropriate follow-up after curative surgery [3–5]. Appropriate follow-up after curative surgery has been proposed by TTR or the accumulative recurrence rate, but not SAR.

The recurrence rate in this study (16.3%) was similar to those in previous studies [3, 7]. The recurrence rate in stage I CC patients was only 1.2%, so we excluded these patients from analysis of TTR and SAR. The recurrence rate in stage I RC patients was 8.4%, and the accumulative recurrence rates were 16.7% and 64.8% at 1 and 3 years, respectively. Therefore, stage I RC patients may require different surveillance from stage II and III patients. We also excluded stage I RC patients for further analysis of TTR and SAR.

Among stage II and III patients, the accumulative recurrence rate was about 50% at 1 year, which was higher than in some previous reports, but similar to that in the FACS trial involving intensive surveillance, including CT [3, 7]. The 2- and 3-year recurrence rates in patients with stages II and III were 70%–80% and 80%–90%, respectively. These are similar to the previous data by Kobayashi et al and suggest that intensive surveillance with CEA checks every 3 months are necessary for at least 3 years, in contrast to the NCCN recommendation of 2 years [3, 14, 15].

TTR differed significantly between CC and RC in stage III patients. Predictors for TTR were different between CC and RC. In CC, patients with high preoperative CEA level and lymphovascular invasion could be followed-up as candidates for early recurrence. On the other hand, predictors in RC were venous invasion and liver metastasis. Therefore, follow-up in RC should include CT to evaluate liver metastasis.

Our data showed that predictors for TTR were not prognostic factors for SAR. Short TTR has been previously reported to influence survival in small cases of study [16, 17]. In this study, recurrence within 1 year was an independent prognostic factor for poor SAR in stage III RC patients. Age (≥75), type of recurrence (M1b), and treatment after recurrence (surgical resection) were also identified as independent prognostic factors for SAR in the current study. Surgical resection was considered a strong prognostic factor for advanced CRC [18, 19]. Recent improvement of treatments against metastatic CRC may provide better SAR than that in current study [20, 21].

Therefore, we proposed that patients with prognostic factors for poor SAR should receive intensive treatment and follow-up after curative surgery. These patients include stage III CC patients with undifferentiated adenocarcinoma or N2 nodal status, stage II RC patients with high preoperative CEA level, and stage III RC patients.

Our study had several limitations. First, it was a retrospective study, and we had no genetic information, such as RAS mutation and microsatellite instability statuses, which are critical genetic markers for prognosis and treatment. KRAS mutation analysis has been available for clinical use in Japan since 2010. Moreover, universal screening for Lynch syndrome was performed in <10% of the hospitals specializing in CRC treatment in Japan [22]. Genetic information was therefore not considered for CRC treatment at the time of surgery in this study. Second, this study also lacked information on the usage of molecular-targeted drugs. Vascular endothelial growth factor and epidermal growth factor antibodies have been used clinically in Japan since 2007 and 2008, respectively. Patients with recurrence should thus have received these drugs for treatment after recurrence. Finally, this was a retrospective study with no rules about follow-up or treatment after surgery, or treatment after recurrence.

Although it seems advisable to detect recurrence as soon as possible to improve the chances of curative resection, the usefulness of intensive follow-up remains controversial. Furthermore, it is unclear if early detection of recurrence could increase the rate of curative treatment and improve survival. Our data demonstrated that short TTR was an independent prognostic factor for SAR in stage III RC patients, who should have received intensive follow-up. However, further prospective studies are needed to confirm our results. Genetic assessment is indispensable in the era of molecular-targeted therapy.

## MATERIALS AND METHODS

### Patients and data collection

This retrospective multicenter study was conducted by the Japanese Study Group for Postoperative Follow-up of Colorectal Cancer (JFUP-CRC). Clinical data were collected for CRC patients who underwent curative surgery at 22 hospitals in Japan between 2007 and 2008. The study was approved by the institutional review board or ethics committee at each hospital and was performed according to the Declaration of Helsinki and Ethical Guidelines for Clinical Research. All patients provided written informed consent. The JFUP-CRC office pooled and prepared the data available for clinical study, as described in the 7^th^ edition of the Japanese Classification of Colorectal Carcinoma [23]. Lymphatic (ly) or venous (v) invasion was classified according to the degree of invasion, as follows: no invasion (ly0/v0), minimal invasion (ly1/v1), moderate invasion (ly2/v2), or severe invasion (ly3/v3). A total of 4992 CRC patients who underwent curative resection in 2007 and 2008, without preoperative chemotherapy/radiotherapy, hereditary CRC, lateral lymph node metastasis, or colitic cancer, were considered suitable for assessment. These patients usually received follow-up with CEA tests every 3 months and CT every 6 months for 3 years, and then CEA every 6 months and CT every 12 months for 3–5 years. The median follow-up time was 72 months in all patients and 60 months in patients with recurrence.

### Data analysis

Differences in clinicopathological factors according to tumor location (CC or RC) were analyzed for all patients and for patients with recurrence. Factors associated with recurrence were assessed using χ^2^ tests.

The influences of clinicopathological factors on relapse-free survival (RFS) and overall survival (OS) in all patients, and TTR and SAR in patients with recurrence were assessed using log-rank tests. Prognostic factors associated with TTR and SAR were subjected to multivariate analyses using a Cox proportional hazard model or logistic analysis. A *P* value of <0.05 was considered significant for all analyses.

## CONCLUSIONS

We recommend that intensive follow-up after surgery is appropriate in stage III CC patients with undifferentiated adenocarcinoma or N2 nodal status, stage II RC patients with high preoperative CEA level, and stage III RC patients.

## Abbreviations

CC: Colon Cancer
CEA: Carcinoembryonic antigen
CRC: Colorectal cancer
CT: Computed tomography
JFUP-CRC: Japanese study group for postoperative follow-up of colorectal cancer
NCCN: National Comprehensive Cancer Network
OS: Overall survival
RC: Rectal cancer
RFS: Relapse free survival
SAR: Survival after recurrence
TTR: Time to recurrence
